# A real-life study of daratumumab-bortezomib-dexamethasone (D-VD) in lenalidomide exposed/refractory multiple myeloma patients: a report from the Triveneto Myeloma Working Group

**DOI:** 10.1007/s00277-023-05443-8

**Published:** 2023-09-20

**Authors:** Gregorio Barilà, Francesca Maria Quaglia, Anna Furlan, Norbert Pescosta, Angela Bonalumi, Chiara Marcon, Anna Pascarella, Martina Tinelli, Elena De March, Albana Lico, Roberto Sartori, Cristina Clissa, Giovanni De Sabbata, Davide Nappi, Marika Porrazzo, Roberta De Marchi, Laura Pavan, Alberto Tosetto, Filippo Gherlinzoni, Mauro Krampera, Renato Bassan, Francesca Patriarca, Gianpietro Semenzato, Renato Zambello

**Affiliations:** 1 Hematology Unit, San Bortolo Hospital, Vicenza, Italy; 2 Hematology Unit, Azienda Ospedaliera Universitaria Integrata Verona, Verona, Italy; 3 Hematology Unit, Santa Maria di Ca’ Foncello Hospital, Treviso, Italy; 4 Ematologia e Centro TMO, Ospedale Centrale Bolzano, Bozen, Italy; 5 Hematology and Transplant Center Unit, Udine University Hospital, DAME, University of Udine, Udine, Italy; 6 Hematology Unit, Ospedale dell’Angelo, Mestre-Venezia, Italy; 7 Department of Medicine, Section of Hematology, Belluno Hospital, Belluno, Italy; 8 Department of Clinical and Experimental Oncology, Onco-hematology Unit, Veneto Institute of Oncology, IOV-IRCCS, Castelfranco Veneto (TV), Italy; 9 Struttura Complessa Ematologia, Azienda Sanitaria Universitaria Giuliano Isontina, Trieste, Italy; 10 Department of Medicine (DIMED), Hematology and Clinical Immunology section, Padova University School of Medicine, Via Giustiniani 2, 35128 Padua, Italy

**Keywords:** Multiple myeloma, Lenalidomide refractoriness, D-VD, Real-life

## Abstract

Treatment of lenalidomide refractory (Len-R) multiple myeloma (MM) patients still represents an unmet clinical need. In the last years, daratumumab-bortezomib-dexamethasone (D-VD) combination was extensively used in this setting, even though only a small fraction of Len-R patients was included in the pivotal trial. This real-life study aimed to evaluate the efficacy and safety of the D-VD regimen in a cohort that exclusively enrolled Len exposed or refractory MM patients. The study cohort included 57 patients affected by relapsed/refractory MM. All patients were previously exposed to Len, with 77.2% being refractory. The overall response rate (ORR) was 79.6% with 43% of cases obtaining at least a very good partial response (VGPR). The D-VD regimen showed a favorable safety profile, with low frequency of grade 3–4 adverse events, except for thrombocytopenia observed in 21.4% of patients. With a median follow-up of 13 months, median progression-free survival (PFS) was 17 months. No significant PFS differences were observed according to age, ISS, LDH levels, type of relapse, and high-risk FISH. Len exposed patients displayed a PFS advantage as compared to Len refractory patients (29 vs 16 months, *p* = 0.2876). Similarly, patients treated after Len maintenance showed a better outcome as compared to patients who had received a full-dose Len treatment (23 vs 13 months, *p* = 0.1728). In conclusion, our real-world data on D-VD combination showed remarkable efficacy in Len-R patients, placing this regimen as one of the standards of care to be properly taken into account in this MM setting.

## Introduction

Over the last several years, multiple myeloma (MM) new treatment options have been engendered at light speed, shifting the treatment paradigm for this condition with a substantial benefit in patients’ survival [1]. In particular, the advent of lenalidomide-based triplet regimens proved to be effective in significantly improving the outcome of relapsed patients with unprecedented overall response (ORR) and progression-free survival (PFS) rates [2–5]. However, the extensive frontline use of lenalidomide (Len) in MM, either as post autologous stem cell transplantation (ASCT) maintenance or in combination with dexamethasone (Len-Dex) as first-line treatment of transplant-ineligible patients [6–8], more and more frequently leads to come across with first relapsed patients who have been exposed or, most importantly, refractory to Len. The treatment of Len refractory (Len-R) patients thereby remains an unmet clinical need [9]. Most patients included in the clinical trials of lenalidomide-based triplet combinations were Len sensitive, while Len refractory cases were generally excluded. In fact, the CASTOR [10] and ENDEAVOR [11] trials, evaluating respectively the efficacy of Daratumumab-Bortezomib-Dexamethasone (D-VD) and Carfilzomib-Dexamethasone (KD) combinations versus Bortezomib-Dexamethasone (VD) alone, included only a limited proportion of Len exposed patients (approximately 36–38%) and an even lower fraction of Len-R cases (24%). Up to now, only the OPTIMISMM study evaluating the efficacy of pomalidomide-bortezomib-dexamethasone (PVD) recruited a high percentage of Len-R patients (71%) with all patients being at least Len exposed [12]. The recent approval in Italy (October 2020) of the PVD triplet, led to the extensive use over the past few years of D-VD and KD combinations in this setting, even though only a small fraction of Len-R patients had been included in the pivotal trial, reporting suboptimal results (median PFS 7.8 months and 8.6 months, respectively) [13, 14].

In this scenario, we conducted a real-life study including only lenalidomide exposed or refractory patients treated with D-VD to evaluate the efficacy and safety of this regimen in this setting of patients.

## Methods

### Study design and patients

This is a retrospective cohort study that included 57 patients affected by relapsed or refractory MM and followed at 10 centers across the Triveneto region (Northeastern Italy) from June 2014 to April 2021. Included patients were anti-CD38 monoclonal antibody naïve and received at least one prior line of therapy including a lenalidomide-based combination. All cases were treated with D-VD regimen according to the approved schedule[10]. Demographic and clinical features, including ISS, FISH analysis, type and symptoms at relapse, were collected.

This study was approved by the Institutional Review Board of Azienda Ospedaliera di Padova (2491P, PD-MM-REG1) and performed according to the Helsinki Declaration. All patients gave their written informed consent before inclusion.

### Statistical analysis

The patients’ demographic, clinical and biological features expressed as categorical variables were compared by Fisher’s exact test. The primary endpoint of the study was progression free survival (PFS) in Len R population defined as the time elapsed between D-VD treatment initiation and tumor progression or death by any cause, with censoring of patients who were lost to follow-up. Secondary endpoints were high quality response rate (≥ very good partial response, VGPR), patient’s overall survival (OS) calculated from the date D-VD treatment initiation to death by any cause or the last known follow-up visit for censored patients, and ≥ grade 3 adverse events rates according to Common Terminology Criteria for Adverse Events (CTCAE) v. 5.0. Prespecified subgroup analyses (age, ISS, LDH, high risk FISH, previous lines oh therapy, type of relapse) were estimated using the Kaplan − Meier method and compared with log-rank test. A univariate Cox proportional hazards regression analysis was employed to evaluate the prognostic relevance of each variable. Results for significant variables were presented as hazard-ratios (HR) and 95% confidence intervals. *P* values < 0.05 were considered significant. Statistical analysis was conducted using Graphpad Prism version 9.4.

## Results

### Clinical and biological features of the study cohort

The study evaluated a cohort of 57 patients (24 female, 33 males) affected by relapsed/refractory MM (Table 1). The median age was 69 years (45–84 years) and 36/57 (63.2%) were aged ≥ 65. Median number of prior lines of therapy was 2 (1–6), with 22/57 (39%) having received ≥ 2 lines of therapy. All patients were anti-CD38 monoclonal antibody naïve and, similarly to the OPTIMISMM study, had received at least a previous lenalidomide based regimen (Len exposed) with 44/54 (77.2%) being Len-R. Considering the type of Len regimen, 13/57 had been previously treated with Len maintenance (22.8%), while in the remaining cases (44/57, 77.2%) Len had been administered in association with dexamethasone alone or in combination with elotuzumab or carfilzomib. Fifty-one out of 57 cases (89.5%) received at least a proteasome inhibitor (PI) based treatment, 10 out of 57 (17.5%) of them being PI refractory. Considering the type of PI, 47/57 cases were previously treated with a bortezomib-based regimen while 19/57 received a carfilzomib-based regimen. A minority of patients received the anti-SLAMF7 monoclonal antibody elotuzumab (3/57, 5.3%) and pomalidomide 6/57 (10.5%). Finally, 43 patients received at least one autologous stem cells transplantation (43/57, 75.4%) during the natural history of the disease.

**Table 1:** Clinical and biological features of multiple myeloma patients’ cohort

Clinical and Biological Features	D-VD treated patients’ cohort (*n* = 57)
Median age (years)	69 (45–84)
≥ 65 years	36/57 (63.2%)
< 65 years	21/57 (36.8%)
Previous lines of therapy	2 (1–6)
1 line	35/57 (61.4%)
≥ 2 lines	22/57 (38.6%)
Len exposed	57/57 (100%)
Len refractory	44/57 (77.2%)
PI exposed	51/57 (89.5%)
PI refractory	10/57 (17.5%)
Double refractory	9/57 (15.8%)
Previous ASCT	43/57 (75.4%)
FISH	30/57 (52.6%)
High risk*	10/30 (33.3%)
Standard risk	20/30 (66.7%)
LDH	
high	5/48 (10.4%)
normal	43/48 (89.6%)
ISS	
ISS I-II	33/46 (71.7%)
ISS III	13/46 (28.3%)
Type of relapse	
Biochemical	25/57 (43.9%)
Clinical	32/57 (56.1%)
Extramedullary disease	6/57 (10.5%)

^*^including t(4;14), t(14;16), del17p

*Len* lenalidomide. *PI* proteasome inhibitor. *ASCT* autologous stem cell transplantation. *ISS* International Staging System

In more than 40% of patients (25/57, 43.9%) D-VD treatment was started following a biochemical relapse while in 32 cases (32/57, 56.1%) a clinical relapse was the reason to begin treatment. Regarding the aggressiveness of relapse, ISS III and high LDH levels were present in 28.3% and 10.4% of cases, respectively. FISH analysis at relapse was available in 30/57 (52.6%) cases and high-risk FISH [including t(4;14), t(14;16) and del17p] was detected in 33.3% of patients. Finally, extramedullary disease has been documented in 6 patients (10.5%).

### Efficacy of D-VD

A median number of 11 cycles was administered in the study cohort (1–35). Responses were evaluable in 54/57 patients, with an overall response rate of 79.6% and 43% of cases obtaining at least a VGPR. In detail, 3/54 patients (5.5%) obtained a minor response (MR), 20/54 (37%) a partial response (PR), 17/54 (32%) a VGPR and 6/54 (11%) a complete response (CR). As for the remaining patients, in 5/54 (9%) a stabilization of the disease (stable disease, SD) was obtained while in 3 cases (5.5%) a progressive disease (PD) was demonstrated. No significant differences in terms of high-quality response rates (≥ VGPR) was observed between Len-R and Len exposed patients (41.9% vs 45.6%, *p* = 0.7542).

Responses were generally fast with a median number of 2 cycles required to obtain at least a PR while best response was reached after a median number of 4 cycles.

Thirty-two patients (56.1%) had discontinued the treatment, mostly due to progressive disease (25/32, 78.1%), followed by toxicities (4/32, 12.5%) and clinicians’ decision (3/32, 9.4%).

### Outcome of D-VD treated patients

**Fig. 1:**
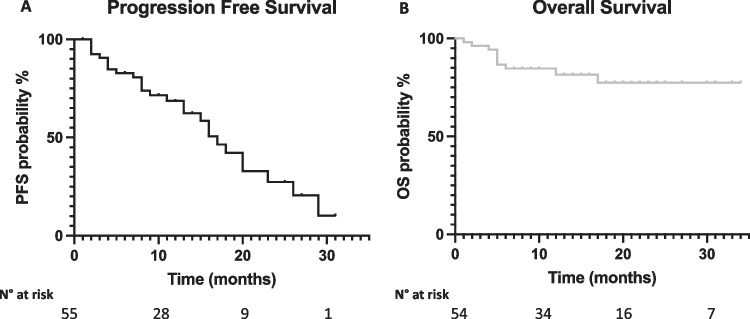
Progression free survival and overall survival of D-VD treated patients. Kaplan–Meier curves showing progression free survival (PFS) (Panel **A**) and overall survival (OS) (Panel **B**) of D-VD treated patients

**Fig. 2:**
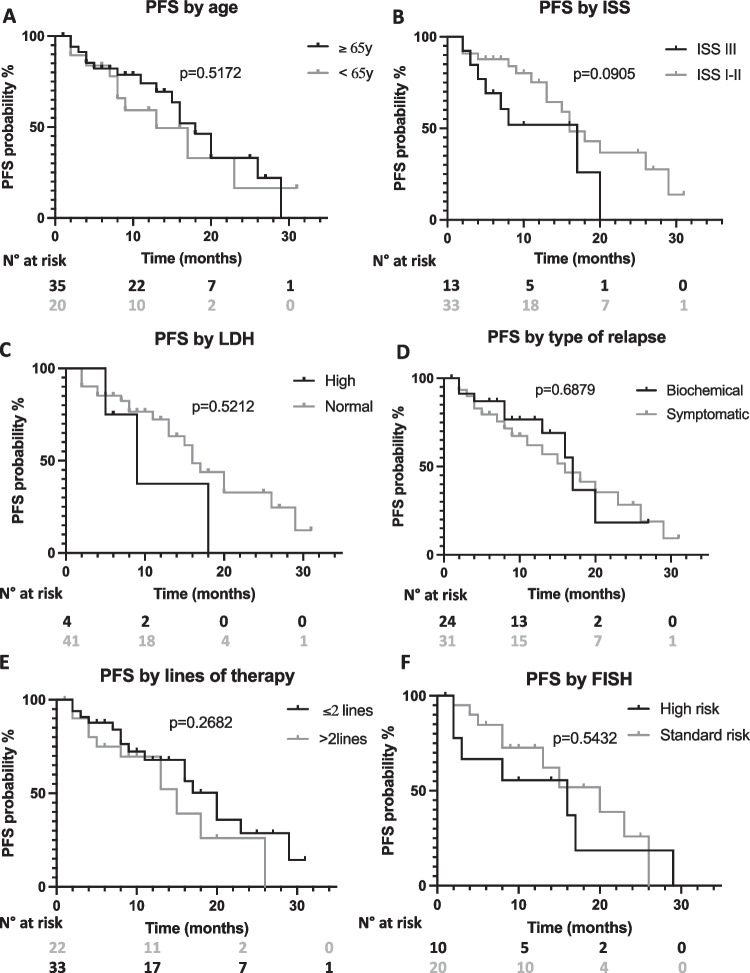
Progression free survival of D-VD treated patients according to clinical and biological characteristics. Kaplan–Meier curves showing progression free survival (PFS) according to age (Panel **A**), ISS (Panel **B**), LDH levels (Panel **C**), type of relapse (Panel **D**), numbers of previous lines of treatment (Panel **E**) and FISH status (Panel **F**). Curves were compared by log-rank test

**Fig. 3:**
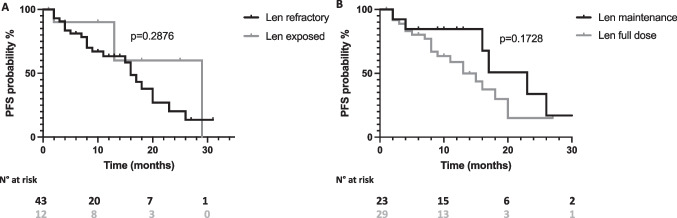
Progression free survival of D-VD treated patients according to Lenalidomide treatment. Kaplan–Meier curves showing progression free survival (PFS) according to Lenalidomide (Len) refractory status (Panel **A**) and previous Len dosage (maintenance vs full dose, Panel **B**). Curves were compared by log-rank test

With a median follow up of 13 months, median progression free survival (PFS) and overall survival (OS) were 17 months and not reached, respectively (Fig. 1, panel A and B, respectively). Univariate analysis results are reported in Table 2. No significant PFS differences were observed according to age (≥ 65 years vs < 65 years, 18 vs 13 months, *p* = 0.5172, Fig. 2, panel A), ISS at relapse (ISS III vs vs ISS I-II, 17 vs 16 months, *p* = 0.0905, Fig. 2, panel B), LDH levels (high LDH vs normal LDH, 9 vs 16 months, *p* = 0.5212, Fig. 2, panel C), the type of relapse (biochemical vs symptomatic relapse, 17 vs 16 months, *p* = 0.6879, Fig. 2, panel D), the number of previous lines of therapy (≤ 2 vs > 2, 20 vs 15 months, *p* = 0.2628, Fig. 2, panel E) or the presence of high risk FISH (high risk vs standard risk, 16 vs 20 months, *p* = 0.5432, Fig. 2, panel F). Regarding previous treatments, Len exposed patients displayed a PFS advantage although not statistical significance as compared to Len refractory patients (29 vs 16 months, *p* = 0.2876, Fig. 3 panel A). Similarly, patients treated after Len maintenance exposure showed a better outcome as compared to patients who had received a full dose Len treatment (23 vs 13 months, *p* = 0.1728, Fig. 3 panel B). Regarding PI exposure, no significant PFS differences were observed between PI refractory and PI sensitive cases (18 vs 17 months, *p* = 0.4747).

**Table 2:** Univariate Cox regression analysis for PFS in D-VD treated multiple myeloma patients

Covariate	HR (95% CI)	*p*-value
Age ≥ 65 years	0.78 (0.35–1.76)	0.5172
ISS III	1.99 (0.73–5-41)	0.0905
High LDH	1.47 (0.36–6.0)	0.5212
Lines of therapy > 2	1.5 (0.67–3.4)	0.2682
Symptomatic relapse	1.2 (0.53–2.55)	0.6879
High Risk FISH	1.31 (0.48–3.57)	0.5432
Len exposed	0.58 (0.24–1.4)	0.2876
Len maintenance	0.55 (0.25–1.3)	0.1728

Table showing the clinical and biological variables evaluated by univariate Cox regression modelling

### Safety

Hematological and non-hematological toxicities are reported in Table 3. Thrombocytopenia was the most frequent adverse event (AE) reported in 26/56 cases (46.6%), with grade 3–4 events in 12 of them (21.4%). Anemia (any grade 28.3%, grade 3–4 3.8%) and neutropenia (any grade 24.1%, grade 3–4 3.7%) were less frequent. Among the non-hematological toxicities, peripheral neuropathy was the most common, being present in 25/56 cases (44.6%), mainly of grade 1–2 (23/56, 41.1%) followed by gastrointestinal and infectious adverse events (17/54 cases each, 31.5%). In detail, infectious complications were mostly respiratory infections (12/17, 70.6%) and in 5 patients (9.3%) a grade ≥ 3 infectious event occurred, including 2 cases of lethal SARS-COV2 infection in patients in CR. On the opposite, cardiovascular AEs involved only minority of patients (5/55, 9.1%).

**Table 3:** Hematological and non-hematological toxicities of D-VD treated patients

Toxicity	Grade 1–2	Grade 3–4
Neutropenia	11/54 (20.4%)	2/54 (3.7%)
Anemia	13/53 (24.5%)	2/53 (3.8%)
Thrombocytopenia	14/56 (25.0%)	12/56 (21.4%)
Peripheral neuropathy	23/56 (41.1%)	2/56 (3.6%)
Hepatic	6/55 (10.9%)	0/55 (0%)
Gastro-intestinal	16/54 (29.6%)	1/54 (1.9%)
Cardio-vascular	4/55 (7.3%)	1/55 (1.8%)
Infectious events	12/54 (22.2%)	5/54 (9.3%)

## Discussion

In a real-life setting we herein demonstrated that the D-VD regimen was associated to high ORR and VGPR rates with a favorable safety profile in previously lenalidomide treated MM patients, thus turning out a reliable therapeutic option in this setting of patients.

In the current therapeutic scenario, almost all MM patients are lenalidomide refractory at first relapse. However, until the recent approval of new triplet regimens including Carfilzomib and Dexamethasone in combination with anti-CD38 monoclonal antibodies Daratumumab or Isatuximab [15, 16] (D-KD and I-KD) or Pomalidomide-Dexamethasone with Daratumumab (Dara-PD) [17], patients’ Len refractoriness represented a major clinical need with few drug combinations available and up to now with limited efficacy. Despite the relevance of this issue in the everyday clinical practice, only the OPTIMISMM trial reliably addressed this problem. The above study showed interesting, even if not exciting, results in Len-R overall populations at first relapse [12], at variance of the less favorable outcomes observed in the small Len-R populations treated with KD or D-VD in pivotal trials [12–14].

In the recent years, real-life studies are becoming increasingly relevant [18–21], as they include unselected patients who are not usually enrolled in clinical trials [22]. In this context, real word data on PVD efficacy are still missing while results of a large study of Len-R patients treated with KD were recently reported [23]. Several retrospective studies evaluated real-life D-VD efficacy, but only few Len-R patients were included [24, 25]. This gap prompted us to conduct this real-world study including the largest cohort reported today of Len-R patients treated with D-VD (*n* = 44). Interestingly our study population was proportionally overlapping with the PVD treated in the OPTISMM trial (100% Len exposed in both studies, Len-R 77.2% vs 71%). The median PFS of the overall and Len-R population herein presented (17 months and 16 months, respectively) were higher as compared to that reported for PVD (11.2 and 9.5 months, respectively). Most importantly, the outcome in Len-R patients was surprisingly improved related to that reported in the pivotal CASTOR trial (16 months vs 7.8 months) [10].

The advent of anti-CD38 monoclonal antibodies in combination with proteasome inhibitors or pomalidomide has significantly improved the outcome of Len-R patients across all trials. The newest combinations of D-KD and I-KD are likely to become the new standard of care for this setting of patients, with the unprecedented PFS of 28.1 months reported in the CANDOR trial [26] and the significant benefit of the triplet regimen in the IKEMA trial as well [27]. More recently, a network meta-analysis of lenalidomide sparing combinations showed that D-VD/D-KD and I-KD regimens have the highest probability to be the best treatments both in Len exposed and in Len-R settings [28]. Most importantly, compelling real word evidence supports the superiority of anti-CD38 based combinations in this setting of patients and real-life D-VD outcomes seem to be better than those shown in the CASTOR trial [24, 25]. Our results obtained in the largest case series available today proved the efficacy of D-VD regimen in Len-R patients thus confirming the appropriateness of the choice of daratumumab based combinations in this difficult to treat MM population.

As mentioned above, given the impressive results of CANDOR and IKEMA trials, KD in combination with anti-CD38 monoclonal antibodies will become the new standard of care for the treatment of Len-R patients. However, it is worth emphasizing that both CANDOR and IKEMA trials included less than 40% of patients Len exposed or refractory (CANDOR 39% and 32%, IKEMA 40% and 32%, respectively [15, 16]. Moreover, the risk of carfilzomib related cardiovascular adverse events as well as the incidence of grade 3–4 infectious complications (> 30% reported in both trials) [15, 16], as compared to < 10% to D-VD trials [10] and in our study, should be taken into account. Finally, the anti-CD38-KD triplet combinations require a higher frequency of hospital admission and an intravenous infusion, at least for Carfilzomib and Isatuximab. In this context, the D-VD regimen provides a completely subcutaneous and quick administration with fewer grade 3–4 infectious and cardiovascular adverse events with a generally manageable safety profile.

In conclusion, notwithstanding the retrospective nature of our study, our real-life experience of D-VD in Len-R MM patients suggests that this combination leads to quick and high-quality responses, with significant PFS and few severe side effects. This regimen should be considered as an optimal candidate therapeutic strategy for Len-R patients, mainly in those ineligibles to D-KD or I-KD due to impaired fitness and/or problems with hospital admission.

## Data Availability

The datasets generated during and/or analyzed during the current study are available from the corresponding author on reasonable request.
